# Co-design methodology for rapid prototyping of modular robots in care settings

**DOI:** 10.3389/frobt.2025.1581506

**Published:** 2025-05-22

**Authors:** Alexandre Colle, Karen Donaldson, Mauro Dragone

**Affiliations:** ^1^ Heriot Watt University, Edinburgh, United Kingdom; ^2^ Edinburgh Centre for Robotics, Heriot Watt University, Edinburgh, United Kingdom; ^3^ CARE Group, The National Robotarium, Heriot Watt University, Edinburgh, United Kingdom; ^4^ School of Engineering, The University of Edinburgh, Edinburgh, United Kingdom; ^5^ The Soft System Group, School of Engineering, The University of Edinburgh, Edinburgh, United Kingdom

**Keywords:** assisted technology, modular robotics, design for additive manufacturing, co-design methodology, care sector

## Abstract

**Introduction:**

This paper introduces a structured co-design methodology for developing modular robotic solutions for the care sector. Despite the widespread adoption of co-design in robotics, existing frameworks often lack clear and systematic processes to effectively incorporate user requirements into tangible robotic designs.

**Method:**

To address this gap, the present work proposes an iterative, modular co-design methodology that captures, organises, and translates user insights into practical robotic modules. The methodology employs Design Research (DR) methods combined with Design for Additive Manufacturing (DfAM) principles, enabling rapid prototyping and iterative refinement based on continuous user feedback. The proposed approach was applied in the development of Robobrico, a modular robot created collaboratively with care home users.

**Results:**

Outcomes from this study demonstrate that this structured process effectively aligns robot functionality with user expectations, enhances adaptability, and facilitates practical integration of modular robotic platforms in real-world care environments.

**Discussion:**

This paper details the proposed methodology, the tools developed to support it, and key insights derived from its implementation.

## 1 Introduction

Health and elderly care systems in many developed nations are increasingly under strain due to ageing populations, creating an urgent demand for innovative technological solutions (Pang et al., 2018; Glasby et al., 2022; Andres, 2022). Robotics in particular has significant potential to improve patient wellbeing and reduce caregiver workload, being already implemented across a range of healthcare applications including surgical operations, rehabilitation, social assistance, and everyday support tasks (Prescott and Caleb-Solly, 2017; Morgan et al., 2022).

However, several challenges hinder the widespread integration of robots into care contexts, notably high costs, technological readiness gaps, and complexities associated with implementation (Houses of Parliment, 2018). Additionally, caregivers and care recipients often have differing perspectives and needs, complicating the development of Assistive Technologies (AT) that satisfy all stakeholders. Further barriers to long-term acceptance include varied perceptions of robotic technologies—while some stakeholders embrace robots for their potential to enhance quality of care, others express concerns over privacy risks, threats to job security, or diminished human interactions (Dragone et al., 2015; Glende et al., 2016; Turja et al., 2020; Peek, 2017; Naneva et al., 2020; Yogeeswaran et al., 2016; McLeay et al., 2021; Maibaum et al., 2022).

Addressing these complex, nuanced requirements necessitates deep user involvement. User-Centered Design (UCD) and co-design methodologies, which actively engage users in shaping technologies, have gained increasing attention within the Human-Robot Interaction (HRI) community (Duque et al., 2019). Yet, despite their potential, few co-design initiatives have produced tangible robotic systems. Typically, robots deployed in care settings are commercial, general-purpose platforms with fixed designs, limiting customisation to software modifications and behavioural adaptations rather than hardware flexibility (Gemeinboeck and Saunders, 2017).

Socially Assistive Robots (SARs) represent a particularly important category within robotics due to demographic projections that foresee older adults outnumbering younger populations by 2050 (Abdi et al., 2018). SARs offer significant potential by improving the quality, frequency, and range of care, supporting not only physical health but also cognitive and social wellbeing of elderly users (Feil-Seifer and Mataric, 2011; Beuscher et al., 2017; Fan et al., 2022). However, the rigid design and manufacturing processes common in current SARs are insufficiently adaptable to meet diverse, evolving user needs.

To overcome these limitations, this paper introduces an iterative and modular co-design methodology that integrates DfAM principles into the development process. This combination enables rapid prototyping, greater adaptability, and enhanced customisation of robotic platforms tailored specifically to users’ unique needs. This methodology is demonstrated through the development of Robobrico, a modular robotic platform created collaboratively with elderly care home users, addressing concerns around cost-effectiveness, adaptability, and usability.

This paper makes the following contributions:

• Introducing a structured, replicable design methodology that integrates DfAM and DR principles.

• Demonstrating the practical application of this methodology through the development of four modular robotic components—Locomotion, Porter, Sanitation, and Social—explicitly designed for elderly care contexts.

• Systematically deriving Functional Requirements (FRs) and corresponding Design Parameters (DPs) using Axiomatic Design, ensuring clear and traceable design decisions.

• Delivering detailed graphical work, including Scenarios, associated FRs, and a breakdown of design phases, all openly accessible via a shared Miro platform to facilitate transparent, collaborative co-design processes.

• Providing a structured evaluation framework, along with key insights, to inform and guide future research and development in modular robotics for elderly care.

## 2 Related work

Disciplines such as engineering and design can intersect to create holistic, user-focused solutions, empowering non-technical audiences to co-develop complex technologies like robotics. From this perspective, co-design emerges as an indispensable strategy for building robotic platforms that are not only technically sound but also adaptable, inclusive, and aligned with the evolving needs of the people they serve. In particular, modular robots hold considerable potential for addressing the demanding requirements of the care sector, yet they remain difficult to access and deploy outside academic settings. DfAM can help overcome these barriers by rapidly producing such platforms, thereby reinforcing co-design as an approach for creating robotic solutions that respond effectively to users’ changing needs. The following section discusses related work, categorized based on topic of interest.

### 2.1 Co-design methodologies

#### 2.1.1 Foundations of design in technological systems

The integration of design into complex technological systems has grown significantly, driven by technology’s expanding societal impact. Similar to engineering, design must consider interconnected system components and integrate user-centered approaches (Mcharek et al., 2019; Buchanan, 2019). Effective design relies on simultaneous consideration of human needs, available technological resources, and business constraints (Tschimmel, 2012). UCD principles—early user involvement, empirical measurement of user interactions, and iterative refinement—remain central to ensuring systems address genuine user requirements (Gould and Lewis, 1985).

#### 2.1.2 Methodologies and tools for capturing user requirements

With users at the core of its inquiry, design as a discipline has developed an extensive array of methodologies to capture user requirements (Baxter et al., 2015a), which can be categorized into various categories.

• Diary studies: Capture users’ information about their activities as they go about in their daily lives.

• Interviews: an interactive and conversational method where a researcher engages with participants verbally.

• Surveys: administering a standardized set of written questions to a large number of participants.

• Card sorts: Guide to inform the decision path on the development of a product.

• Focus group: A group give feedback on a common project.

• Field studies: Research enquiry on site, within users’ environments.

In complement to different methods, researchers can employ the Wants and Needs (W&N) method to be used as a quick and valuable tool in interviews. The W&N method is structured around three distinct types of questions, as proposed by Baxter et al. (Baxter et al., 2015b): what users want and need, what users anticipate and what do they seek in a system.

Dedicated co-design tools provide structure and clarity in collaborative environments, enabling idea exchange and cohesive team alignment (Axelsson et al., 2022). Agile methodologies, which emphasize flexibility, iterative cycles, rapid prototyping, and continuous user feedback, are particularly suited to robotics development due to frequent requirement adjustments (Kazakevich and Joiner, 2023). This iterative approach facilitates real-time feedback integration and development of tangible outcomes through Minimum Viable Products (MVPs), systematically advancing projects while maintaining user-centeredness (Easterday et al., 2018).

**FIGURE 1:**
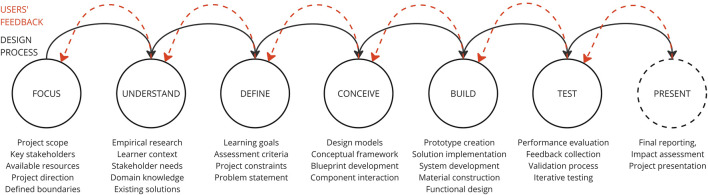
Phases of the DR methodology, summarising the iterative co-design process foundational to this paper.

Figure 1 summarises this iterative DR methodology, clearly illustrating the structured co-design approach employed in this study.

#### 2.1.3 Frameworks and applications in co-design and robotics

HRI increasingly values multidisciplinary and user-centered approaches over technology-led, isolated solutions (Bartneck et al., 2020; Breazeal et al., 2016). Methods such as co-design, co-production, and co-creation actively involve users throughout technology development, significantly improving acceptance, relevance, and long-term usability of social robotics (Hallam-Bowles et al., 2022; Ostrowski et al., 2021; Cagiltay et al., 2020; Azenkot et al., 2016).

Such methodologies facilitate broad user involvement, significantly enhancing trust, ownership, and ultimately adoption by directly integrating user concerns and feedback into designs (Søraa et al., 2022; Mincolelli et al., 2019; Duque et al., 2019). These benefits are demonstrated in various contexts, from supporting older adults (Ostrowski et al., 2021; Lee et al., 2017) to assisting visually impaired users (Azenkot et al., 2016) and developing user-centered robotic applications (Tonkin et al., 2018; Winkle et al., 2021). Although predominantly focused on improving interactions with existing robot platforms, very few co-design frameworks systematically address the complexities of physical robot development and manufacturing challenges (Sumner et al., 2021).

Notable exceptions include educational and outreach-focused designs such as YOLO (Alves-Oliveira et al., 2019) and Opsoro (Darriba Frederiks et al., 2019), although these cases lack replicable frameworks for systematic implementation.

#### 2.1.4 Challenges and future directions in co-design for robotics

Despite its advantages, co-design in robotics faces significant resource demands, complexity, and cost barriers, often limiting projects to behavioural or interface design rather than comprehensive robotic systems (D’Onofrio et al., 2018). Standardized methods and structured development frameworks are needed to systematically support implementation, commercialisation, and broader adoption of robotic technologies, particularly in care settings (Axelsson et al., 2022; Alves-Oliveira et al., 2022).

To address diverse and evolving user needs, modular and adaptable design solutions have gained importance. Modular robots offer the flexibility to accommodate shifting user requirements over time, providing tailored solutions in healthcare contexts (D’Onofrio et al., 2018; Zou et al., 2022; Bedaf et al., 2016; Abdi et al., 2019).

For instance, Axelsson et al. (Axelsson et al., 2022) propose a structured but linear “Design Path” model, beneficial for clearly defined stages but lacking the iterative responsiveness of Agile methodologies. In contrast, Axiomatic Design supports modular decomposition of complex problems into manageable FRs and DPs, ensuring efficient and structured refinement and implementation (Goel and Pirolli, 1992; Brown et al., 2020).

**FIGURE 2:**
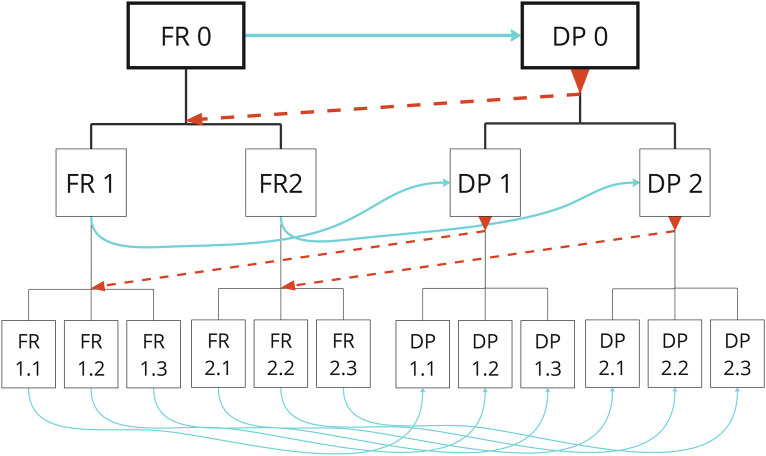
Hierarchical relationship between FRs and DPs illustrating iterative design flow (Brown et al., 2020).

Within this study, simplifying this method by initially focusing on essential FRs and DPs provides clarity and a systematic approach for iterative refinement and physical design outcomes (Figure 2), ultimately supporting the development of effective robotic solutions deployable outside controlled laboratory environments.

Acknowledging the complexity and individuality of user needs, modular robotics design emerges as a promising approach to creating flexible, user-friendly, and affordable solutions tailored to diverse requirements in care contexts.

### 2.2 Modular robots

#### 2.2.1 Introduction and foundations

Co-design approaches in care robotics highlight the necessity of addressing diverse user requirements. Modular robots offer a promising solution by adapting functionality to specific user contexts rather than deploying multiple single-purpose robots. Modular robots consist of interchangeable modules, each transmitting power, force, and communication through standardised interfaces (Wei et al., 2012). These systems are categorised as either *homogeneous*, where identical modules change functionality through shape reconfiguration (Aloupis et al., 2009), or *heterogeneous*, where distinct modules perform specialised functions requiring advanced task and configuration planning (Ahmadzadeh et al., 2016).

#### 2.2.2 Classification and characteristics

Modular robots typically fall into four classifications (Ahmadzadeh et al., 2016): (i) Fixed-Configuration robots with static arrangements; (ii) Manually Reconfigurable robots, offering flexibility through human intervention; (iii) Self-Reconfigurable robots, capable of autonomous morphological adjustments; and (iv) self-replicable robots, autonomously gathering and reassembling modules (Kriegman et al., 2020; Bianco and Nolfi, 2004). Each type presents trade-offs regarding complexity, autonomy, and practical deployment.

Recent standards, such as the ISO 22166-1:2021 aim to improve modular robot interoperability, emphasising seamless power/data connections, open interfaces, and module reuse to enhance efficiency, adaptability, and reduce costs (Zou et al., 2022).

#### 2.2.3 Technical approaches and challenges

Achieving modularity can also involve software frameworks for robot-environment interaction, such as PEIS-Ecology (Saffiotti et al., 2008), though these remain largely untested in real-world contexts due to complexity in interoperability and self-configuration. Stand-alone modular platforms, while simpler in deployment, face substantial resource, regulatory, and manufacturing barriers that hinder widespread commercial adoption (Zou et al., 2022).

Substantial advances in Industry 5.0 or a more human centric version called 4.0S by Raja et al. (Raja Santhi and Muthuswamy, 2023) and the digital transformation of manufacturing are currently enabling more flexible production practices (Pansare et al., 2022; Dilberoglu et al., 2017). These developments stem from increasing demands for customisation and modularity, supported by technologies that accommodate smaller production batches, human-centered design, and accelerated technical development. However, significant investment in infrastructure and skills, coupled with entrenched manufacturing processes, have limited practical adoption, particularly in healthcare robotics.

#### 2.2.4 Practical applications and future directions

Modular robots distribute complexity across specialised modules, providing flexibility over purely multifunctional systems. For instance, Care-O-Bot, an intelligent mobile robot designed for elderly care at home, smoothly performs complex tasks like mobility assistance or fetching objects, yet it remains susceptible to failures from minor environmental variations (Reiser et al., 2013). The authors suggest prioritising simpler yet practical functionalities, highlighting modularity as a key solution to adaptability and robustness within diverse caregiving scenarios.

In research contexts, modular robots offer significant advantages due to their adaptability and scalability, enabling versatile task execution in extreme or dynamic environments. The design principles behind modularity are also transferable to broader fields, amplifying their potential impact beyond specific research applications. Linner et al. (2025) introduced the Multi Robotic Assistant System (MRAS), a ceiling-mounted modular robot aimed at assisting elderly users by integrating environmental controls, air purification, medication dispensing, and mobility support. They emphasised the value of Plug-n-Play modularity to promote user independence and reduce reliance on technical support (Gibson et al., 2019). However, MRAS lacks systematic user involvement in its design process, highlighting the need for more robust user-centred methodologies to ensure the robot effectively addresses actual user priorities.

Similarly, Fable, a modular social robot intended for educational use, allows non-experts to customise anthropomorphic shapes and interactions via interchangeable modules (Magnússon et al., 2025). Despite its modularity, the development of Fable did not incorporate participatory design methods. In contrast, Quori, an affordable modular social humanoid robot, utilised user inputs through surveys, workshops, and community engagement within the Human–Robot Interaction field to inform its modular appearance (Specian et al., 2022). Nevertheless, Quori’s modularity primarily impacts aesthetics rather than core functionalities. The SMOOTH-robot employed co-design to address user requirements through modularity, accommodating logistics, guidance, and beverage delivery functions (Krüger et al., 2021). Despite the successful integration of user input, traditional manufacturing processes limited iterative development and design flexibility, restricting ongoing modifications based on user feedback.

As user needs for socially assistive robots continually evolve, modularity and user-driven adaptation become increasingly important. Gibson’s concept of “Bricolage” emphasises user empowerment to continuously adapt technology according to their evolving requirements (Gibson et al., 2019). Thus, modular robotics should incorporate ongoing user feedback to allow iterative improvement and user ownership of technological solutions.

However, the cost of developing research-focused modular robotic platforms typically hinders widespread commercial application. To address this, exploring alternative prototyping and manufacturing techniques that reduce expenses and enhance accessibility is vital. Such strategies could enable practical and economically feasible applications of modular robotic systems.

### 2.3 Additive manufacturing

Manufacturing industries increasingly shift from mass production towards mass customization due to rising consumer demand for personalized products and services (Theilmann and Hukauf, 2014; Ameen et al., 2021). Traditional manufacturing methods, however, remain limited by part complexity, scale constraints, and sustainability concerns (Mittal and Sangwan, 2014; Badhotiya et al., 2022). In response, Industry 4.0 and Industry 5.0 paradigms emphasise digitalisation, decentralisation, sustainability, and human-centric customisation, necessitating advanced manufacturing techniques that can efficiently handle personalised production demands (Xu et al., 2021; Maddikunta et al., 2022). In this context, DfAM provides a compelling alternative, enhancing sustainability (Colorado et al., 2020), manufacturability, reliability, and cost-efficiency by directly fabricating complex products from digital models (Tang and Zhao, 2016).

DfAM, when integrated within Design for X methodologies (Kuo et al., 2001), particularly benefits robotic system development by facilitating mass customization, on-demand production, and improved material utilization. These capabilities shorten lead times, lower inventory demands, and support decentralised manufacturing (Attaran, 2017). Parametric Modelling (PM), a key feature in contemporary CAD tools, complements DfAM by automating adjustments across complex, interlinked components, further streamlining the design and development process (Camba et al., 2016; Madrigal and Jeong, 2022).

#### 2.3.1 A framework to support DfAm for robots

**FIGURE 3:**
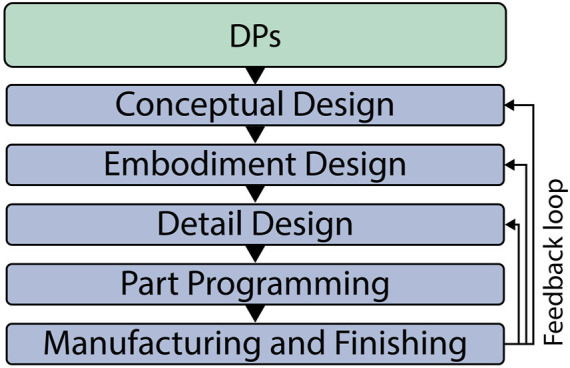
Simplified steps of the DfAM framework by Pradel et al. (2018), applied to the Robobrico project during the BUILD stage of the DR process.

A practical framework for leveraging DfAM in product development is presented by Pradel et al. (Pradel et al., 2018). This structured approach (Figure 3) maps DfAM principles onto each design stage, from initial concepts to manufacturing, simplifying knowledge application and bridging the gap between design intent and manufacturability. By highlighting specific AM-guidance early, this framework supports informed decision-making and encourages innovation. By establishing a feedback loop, this process enables continuous iterations, allowing improvements to be made at each stage of the manufacturing workflow.

Combining user-centered methodologies with DfAM enables rapid development of modular robotic prototypes, encourage active end-user involvement and validation throughout the design process. Such integration enhances the likelihood of achieving functional, adaptable robots that effectively meet stakeholders’ needs.

## 3 Design research methodology

Previous sections highlighted the importance of establishing a systematic methodology for developing modular and customisable robots, while simultaneously measuring and ensuring sustained user engagement throughout the design process. Such a methodology should enable designers and roboticists to collaborate closely with end-users, effectively capturing and addressing their diverse requirements and contexts.

In defining our methodological approach, several established frameworks commonly used in robotics and product development were considered, including Agile methodologies (Kazakevich and Joiner, 2023; D’Onofrio et al., 2018), UCD (Gould and Lewis, 1985; Baxter et al., 2015c; Norman, 2013), and Lean User Experience (Lean UX) (Seiden and Gothelf, 2013; Tonkin et al., 2018). Agile methodologies are valued for their rapid prototyping and iterative cycles, facilitating quick responses to user feedback but typically prioritising short-term incremental improvements over broader conceptual and foundational insights (Kazakevich and Joiner, 2023). Conversely, UCD offers a structured, user-focused approach but often lacks the explicit iterative structure to adapt quickly to evolving technical constraints or novel user needs emerging throughout the process (Baxter et al., 2015a). Lean UX emphasizes rapid validation of hypotheses through continuous prototyping; however, it is primarily oriented toward digital interfaces and incremental software improvements rather than the holistic integration of hardware, software, and user interactions inherent to robotics (Tonkin et al., 2018; Seiden and Gothelf, 2013). Given these limitations, none of these frameworks individually satisfied the project’s comprehensive requirements for sustained user involvement, modular flexibility, and systematic evaluation at each design stage.

Initially, the Double Diamond (DD) framework (Alves-Oliveira et al., 2019; Bardaro et al., 2022; Ostrowski and Breazeal, 2022) was considered due to its popularity, its use in robotics projects, as in clearly communicating design processes to stakeholders outside the design discipline. However, its linear progression—structured around the four stages of *Discover*, *Define*, *Develop*, and *Deliver*, limited opportunities for iterative user engagement and incremental evaluation (Banbury et al., 2021). The DD framework’s rigidity and the absence of built-in mechanisms for continuous feedback at intermediate stages proved inadequate for addressing the dynamic nature of HRI of this project, where iterative refinement, adaptability, and ongoing evaluation of user acceptance and usability are were essential.

To overcome these constraints, the step-by-step DR methodology proposed by Easterday et al. (Easterday et al., 2018) was adopted. This approach provided a structured, modular, and explicitly iterative framework, enabling flexibility to adapt to changing user needs and technological developments over the project lifespan. DR’s inherent modularity facilitated systematic evaluation at each stage, allowing continuous integration of user feedback, direct traceability of design decisions, and clear alignment between FRs and DPs following Axiomatic Design principles. This ensured robust user-centred outcomes alongside a clear developmental roadmap that maintained user input at its core throughout each phase of the process.

This methodology was implemented over 1 year, enabling systematic end-user co-design and rapid prototyping of a basic yet fully functional robotic system. Due to initial COVID-19 restrictions, digital ideation sessions were conducted using the Miro platform (Alexandre, 2024), later transitioning to in-person workshops as restrictions eased. The DR approach adopted here, summarised in Figure 1, comprises seven structured phases, FOCUS, UNDERSTAND, DEFINE, CONCEIVE, BUILD, TEST, each described in detail in the following sections. At the conclusion of each phase, a summary highlighting key components is provided to clarify and guide the development process.

### 3.1 Focus the problem

The FOCUS stage establishes the scope, project direction, key stakeholders, and resource availability. This initial phase ensures that the team identifies a clear, achievable goal, aligns stakeholder interests, and defines team responsibilities, laying a foundation for effective collaboration in subsequent DR stages.

#### 3.1.1 Design planning

To initiate the study, a suitable care-sector partner was identified through institutional outreach, resulting in a collaboration with a care home in Edinburgh. Ethical approval was obtained from the Engineering and Physical Sciences Ethics Committee at Heriot-Watt University (Ref 2020-0474-2638).

The care home management expressed strong interest in innovative robotic solutions to enhance care provision and resident engagement. The onset of COVID-19 further underscored robotics’ potential for enabling safer interactions. The management facilitated participant recruitment, involving two volunteer residents, two care managers, and one property manager, each providing unique insights into operational and environmental contexts. To mitigate preconceived notions about robotics (Furlough et al., 2021), the project was framed broadly as Assistive Technology (AT), enabling participants to share genuine experiences without unrealistic expectations.

Acknowledging the limited sample size, this decision aligns with Nielsen’s usability guidelines (Nielsen, 2000), which indicate that five users can effectively uncover most usability issues, with diminishing returns observed in larger groups. Baxter et al. (Baxter et al., 2015d) similarly support small participant numbers for achieving deeper qualitative engagement and trust. Furthermore, Salomé et al. (Salomé et al., 2025) confirm that small groups are appropriate for early-stage participatory co-design with older adults, providing substantial qualitative insights through iterative engagement. The research team, comprising one HRI PhD student, one illustrator, and two roboticists, leveraged this small-scale, focused approach to develop this research.

#### 3.1.2 Design referencing system

Axiomatic Design provided the continuous reference framework, structuring the hierarchical relationship between FRs and DPs, as illustrated in Figure 2. At the highest level, FR0 was defined as improving the care experience, while DP0 represented robotics as a broad solution. Subsequent phases involved iterative refinement and prioritisation of specific FRs and DPs.

#### 3.1.3 Focus phase summary

• Project Scope: Improve the care experience in a residential care home, by exploring the potential for an AT solution with a focus on robotics.

• Key Stakeholders: Partnership was established with a care home in Edinburgh, engaging residents, care managers, and property staff; research team consisting of an HRI PhD student, one illustrator, and two roboticists.

• Available Resources: Access to the care home, its staff and residents, institutional support from Heriot-Watt University, and a small interdisciplinary team.

• Defined Boundaries: Focus on user-driven insights and minimal viable functionality in response to staff and resident needs; maintain to feasible technology due to limited resources and limiting participant numbers to ensure deep engagement and meaningful insights with limited team.

### 3.2 Understand the problem

The UNDERSTAND stage focuses on capturing and analysing critical information about the problem domain, context, stakeholder needs, and previously attempted solutions. Researchers conduct empirical studies, such as observations, interviews, and surveys, and review secondary sources to synthesise insights into structured outputs. These outputs, including reports, thematic analyses, and design tools like Personas, provide guidance for subsequent stages in the DR process.

#### 3.2.1 The problem space

Initially, research focused on understanding current implementations and perceptions of SARs within care environments (Colle et al., 2021). Despite the optimism around AT, complexities in robotics and assisted-living devices often hinder user adoption. Gibson (Gibson et al., 2019) observes that caregivers frequently modify or “tame” AT to align with simpler daily tasks than originally designed for. This process highlights the critical importance of deeply understanding end-user requirements, fostering both trust in the intervention and confidence in its developers. Reflecting this insight, the project was named Robobrico, inspired by Gibson’s notion of Bricolage, meaning “Do it Yourself.”

Following participant recruitment, an introductory meeting took place at the care partner’s office, providing an opportunity for all parties to familiarise themselves with each other’s roles and the project’s objectives. Semi-structured online interviews were conducted to collect demographic data and document the Technology Experience Profile (TEP) of each participant (Table 1). The five participants had no prior experience with robotics but demonstrated proficiency in mainstream technologies such as teleconferencing tools and the Miro online workspace.

**TABLE 1:** Demographics of participants, including professional status, education, ethnicity, and technology experience.

Factor	Category	Count
Professional Status	Retired with volunteering	2
	Active	3
Education	Professional Degree	1
	Bachelor’s Degree	1
	College Degree	2
	Master’s Degree	1
Ethnicity	White (Scottish & British)	4
	Asian other	1
Technology Experience	Daily usage of technology	5
General Concerns	Social isolation	
	Pain and mobility	
	Engaging older tenants digitally	
	Supporting older adults’ independence	
Hope about Technology	Easy connection with family members	
	Better pain management	
	Improved technological adoption	
	Better support via technology	

#### 3.2.2 Observational study

To capture user needs and preferences in depth, a selection of design methodology tools was carefully selected. Given the qualitative depth required and the practical constraints of COVID-19, methods such as semi-structured interviews, Personas, and Journey Mapping were chosen. Methods typically focusing on larger-scale quantitative data, such as surveys, or those prioritising group consensus, like traditional focus groups, were intentionally excluded. Similarly, although initial on-site observations informed spatial constraints, extensive longitudinal field studies were beyond the project’s scope and timeline.

Initial semi-structured interviews provided detailed insights into participants’ backgrounds, technology experiences, and attitudes toward assistive robotics, establishing foundational knowledge for subsequent activities.

Personas were developed to synthesise key user types from qualitative data, offering participants a relatable yet comfortable means of exploring user experiences without directly referencing personal situations (Baxter et al., 2015e; Brenner and Uebernickel, 2016). Each participant created two Personas, one carer and one resident, using a structured approach resembling diary studies (Baxter et al., 2015a), systematically capturing their ideation progress.

A total of eight distinct Personas emerged, evenly divided between residents and caregiving staff, reflecting diverse profiles in mobility, cognitive abilities, and social engagement. Staff Personas depicted a busy but compassionate caregiving team with varying interests in technology. Notably, one persona pair included a semi-retired 70-year-old father with limited mobility, cared for by his daughter, effectively covering a broad spectrum of user needs. Another illustrative example, “Mary,” is a 50-year-old nurse from Edinburgh with moderate technology familiarity, whose daily frustrations with technology—such as difficulties with a malfunctioning printer—captured authentic user challenges.

Following persona development, the Journey Map tool from the Service Design Tool platform (ObloDesign, 2023) enabled participants to reconstruct detailed scenarios of typical daily interactions. Chosen for its intuitive structure and comprehensive scope, Journey Mapping transformed abstract experiences into clearly articulated, structured design requirements (Miaskiewicz and Kozar, 2011). Due to its detailed nature, the Journey Mapping process was divided into three separate sessions per participant, ensuring adequate engagement and task completion. It was structured into distinct categories representing various aspects of daily life, as summarised in Table 2.

**TABLE 2:** Overview of the main sections of the Journey Map tool used to reconstruct and analyse a typical day in the life of a persona.

Section	Description
Scenario	Participants were asked to create a scenario for a specific “Use Case” and assign a task to their Personas. Scenarios are valuable in design because they provide concrete, story-based representations of how a persona might interact with a product or system in a specific context (Baxter et al., 2015a)
Positive and Negative Aspects	The “Journey Map” method is similar to “Customer Journey Mapping.” Both capture user experiences within a specific context. While Customer Journey Mapping tracks emotional experiences (positive or negative) (Crosier and Handford, 2012), this section focuses on comparing positive and negative experiences. Participants provided additional context to inform their subsequent inputs in the Journey Mapping process
Human Role	Using the Wants and Needs (W&N) method, participants identified the characteristics a human should have to support their persona in the scenario
Robot Role	Reflecting on the human role, participants explored tasks a robot could perform. Using the W&N method, they described the FRs for a robot to assist their persona or perform tasks without human involvement. Participants were encouraged to ignore technological limitations and use imagination to guide their responses

Scenarios and FRs generated from this activity were documented using clear illustrations, chosen for their effectiveness in conveying information and sustaining participant engagement during workshops (Murchie and Diomede, 2020). Different visual styles and colours distinguished scenarios (warm, relatable graphics) from FRs (clear, schematic visuals) to improve participant comprehension and retention.

**FIGURE 4:**
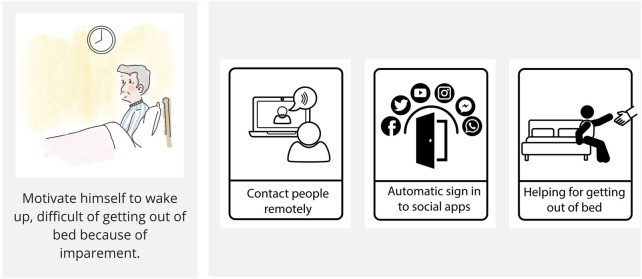
A user scenario (left) illustrates an event affecting a persona, while the co-defined FR1–FR3, (right) focus on participants requirement for that particular scenario.

The first workshop yielded 41 distinct scenarios derived from Personas, leading participants to collaboratively define 98 potential robotic FRs. These outputs are extensively documented in Supplementary Materials accessible via the shared Miro platform (Alexandre, 2024). An example scenario and its associated FRs are illustrated in Figure 4, showcasing a retired older gentleman experiencing mobility issues, prompting participants to define specific robotic functionalities (FR1, FR2, FR3).

#### 3.2.3 Understand phase summary

• Empirical Research: Data was gathered through interviews, observational studies, and design workshops to uncover user needs, environmental constraints, and attitudes toward assistive robotics in care contexts.

• Learner Context: Personas and Journey Maps were used to model users’ day-to-day experiences, highlighting key moments where robotic assistance could support autonomy, social engagement, and wellbeing.

• Stakeholder Needs: Participants, both residents and caregivers, identified FRs through guided co-design activities, resulting in 98 user-informed design goals.

• Domain Knowledge: Insights from healthcare and AT literature informed design constraints, including spatial considerations, emotional barriers to adoption, and known challenges with technological interfaces.

• Existing Solutions: Through literature review, the team critically examined relevant ATs, noting limitations in adaptability, personalization, and user trust—issues the Robobrico project aims to address.

### 3.3 Define goals

The DEFINE phase helps to specify the problem clearly, setting the precise goals, assessment criteria, and research objectives for the project. The primary aim is to transform an indeterminate problem—one without an immediate solution—into a determinate problem with clearly defined parameters. This process refines abstract user insights gathered in the previous phases into actionable FRs, preparing them for practical design development.

**FIGURE 5:**
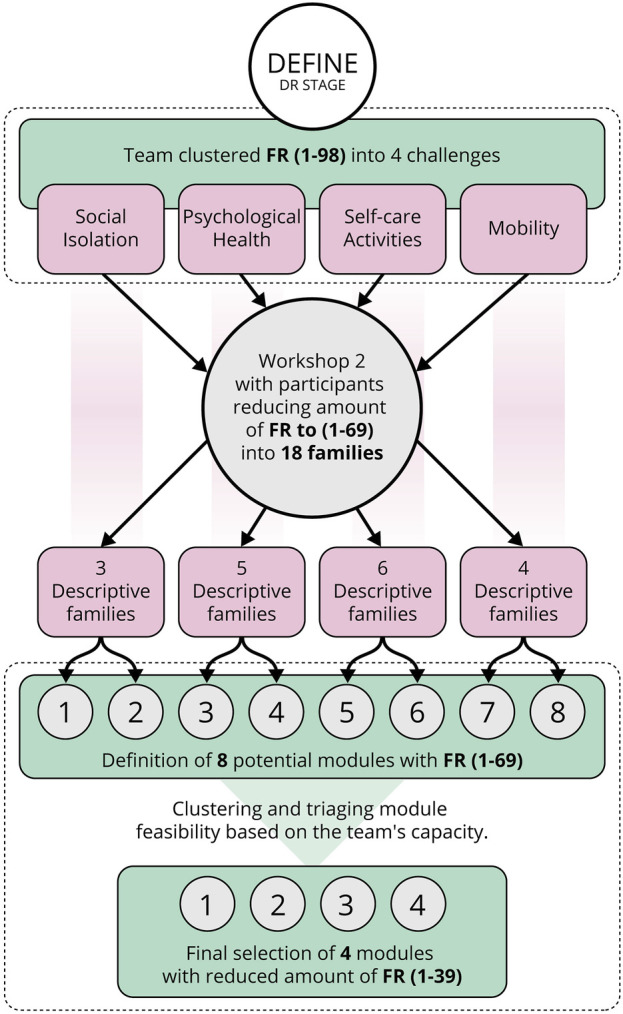
Process flowchart illustrating the critical stages of the step-by-step approach used for clustering and refining FRs during the DEFINE stage.

To achieve this, participants were systematically guided to identify, prioritise, and cluster the top-level FRs (numbered 1–98) based on their Personas’ identified needs. A qualitative clustering method (Brickey et al., 2010) facilitated this process, allowing participants to group key functionalities essential to their Personas while drawing from their own experiences and knowledge. This stage effectively operationalised abstract user needs, clearly defining the scope for practical implementation in subsequent design stages. To systematically approach this clustering and refinement, the DEFINE phase followed a structured step-by-step method, progressively reducing complexity as illustrated in Figure 5.

#### 3.3.1 Clustering FRs

Initially, the FRs (1–98) identified by participants were grouped into four key areas representing common challenges faced by older adults, based on established categories from the literature (Bedaf et al., 2014; Abdi et al., 2019). This structured the complex array of FRs, providing participants with a clearer context for prioritisation. The four challenges are:

1) Social Isolation: Focusing on encouraging social interactions and combating loneliness.
2) Psychological Health: Supporting mental health and providing psychological support.
3) Self-Care Activities: Maintaining independence in daily self-care tasks.
4) Mobility: Addressing mobility issues and enhancing independent movement.

#### 3.3.2 Ranking and categorizing FRs

After clustering the initial 98 FRs into four broad challenge areas, a second online workshop was held via Miro to refine and prioritise each FRs. During this 2-h session, participants revisited each FR’s importance in light of the Personas established earlier. The evaluation followed three core criteria: (i) User Relevance, which elevated FRs aligned with participants’ daily tasks; (ii) Uniqueness and Clarity, consolidating or discarding items deemed redundant or ambiguous; and (iii) Reflective Insights, leveraging the interval since the previous workshop for deeper reflection on earlier decisions.

This refinement process resulted in the elimination of less critical and redundant FRs, reducing the original set from 98 to 69. These remaining FRs were grouped into 18 descriptive families, each clearly defined to represent distinct and conceptually coherent subsets of the four challenges (see Table 3). These descriptive families provide context and structured guidance for subsequent module development by making explicit the underlying user priorities and themes pertinent for SARs module development.

**TABLE 3:** FR (1–69) were grouped into 18 descriptive families, each followed by a brief explanation to clarify the scope and intent of the grouping.

Descriptive families	Description
1. Social Isolation	
Assisted Remote Communication	Helping users communicate with family and friends remotely, with simplified interfaces for video calls and messaging
Automated Chat	Providing conversational interaction to reduce loneliness, possibly through AI chatbots or voice assistants
Technical Support	Assisting users with technical issues or using devices, offering guidance or troubleshooting help
2. Psychological Health	
Sleeping Support	Aiding users in establishing healthy sleep routines, possibly through reminders or relaxation techniques
Music Player	Playing music to enhance mood or provide entertainment, potentially personalized to user preferences
Mental Health Assessment	Monitoring psychological wellbeing and alerting caregivers if concerns are detected
Entertainment	Providing games or activities to stimulate cognitive function and enjoyment
Reminder	Offering reminders for medications, appointments, or daily activities to support memory
3. Self-Care Activities	
Practical Tech Support	Assisting with the use of everyday technology devices to enhance independence
Carer Support	Supporting caregivers in their tasks, potentially reducing their workload
Healthcare Support	Assisting with health-related tasks, such as monitoring vital signs or medication management
Support House Chores	Helping with household tasks to maintain a clean and safe living environment
Interactive Medical Support	Providing remote assistance during medical emergencies or routine care
Physical Diagnostics	Provide with the ability to assist with vital and visual assessments
4. Mobility	
Autonomous Medication	Delivering medications to the user at scheduled times without human intervention
Carry Objects Around	Assisting in transporting items within the home to reduce physical strain
Physical Support	Providing support for mobility, such as helping users stand or walk safely
Carry and Provide Drink and Food	Bringing meals or beverages to the user, enhancing convenience and safety

#### 3.3.3 Organising FRs into potential robotic modules

Given the diversity of user requirements and the practical limitations identified earlier, a modular robotic approach was adopted. Modular robots, as discussed in the literature, offer adaptability, scalability, and customisation capabilities, making them particularly suited to addressing diverse and evolving user needs within resource constraints. Specifically, a Manually Reconfigurable robot type was selected, allowing non-technical users to interchange modules easily, maintaining simplicity and practicality for care environments.

Using the 18 previously defined descriptive families, the team collaboratively organised the 69 FRs into preliminary robotic modules via the Miro platform (Colle, 2025a), documented in the Supplementary Materials. This categorisation involved identifying common functionalities, overlaps, and potential integrations, leading to an initial set of eight modules: Social, IoT, Sanitation, Medication Dispenser, Porter, Physical Support, Hospitality, and Health. For instance, within the Social module, FRs such as *Playing Relaxing Music* and *Contacts People Remotely* were grouped together to reflect participants’ prioritisation of social engagement and emotional support functionalities. Some modules exhibited natural interdependencies—for example, functionalities of the Medication Dispenser module overlapped significantly with those in the Social module, suggesting opportunities for consolidation.

The process clarified the design problem space, highlighted functional interdependencies, and enabled the identification of feasible modules aligned with project constraints.

#### 3.3.4 Assessing feasibility of clustered FRs

After defining the initial set of eight modules; Social, IoT, Sanitation, Medication Dispenser, Porter, Physical Support, Hospitality, and Health, a detailed feasibility assessment was conducted on each of the 69 FRs within these modules, explicitly considering technological readiness, available resources, and the expertise within the team. The feasibility evaluation categorised each FR into one of three distinct groups, available in detail on the Miro board provided in the Supplementary Documents (Colle, 2025a):

*(i) Possible Now* (39 FRs across four modules): functionalities immediately implementable with existing resources and expertise; *(ii) Future Development* (22 FRs across five modules): functionalities requiring technological advancement or specialised knowledge currently beyond the team’s capability; *(iii) Possible with Financial Support* (Eight FRs across three modules): functionalities achievable with additional financial investment or external support. For instance, the FR “Performing a visual health check” was categorised under Future Development due to its technological complexity exceeding current team capabilities. Conversely, “Dispensing medication during the day” fell under Possible with Financial Support, as it needed specialised equipment currently beyond the project’s financial scope.

#### 3.3.5 Final modules selection

Based on the feasibility assessment described previously, the project scope was deliberately constrained to the *(i) Possible Now* category. This decision refined the initial eight modules down to four, each aligned directly with both user priorities and practical project constraints. Selecting modules that could be immediately developed ensured that end-user requirements would be effectively addressed within existing team resources and expertise. The final modules selected were as follows:

• Social module: Incorporating functionalities related to reminders, entertainment, music playback, assisted remote communication, automated chat, and healthcare support—addressing social interaction and psychological health needs.

• Sanitation module: Supporting essential household chores to maintain cleanliness and a safe environment—critical for self-care and health maintenance.

• Hospitality module: Enhancing convenience and safety by providing assistance with serving and carrying food and beverages—directly responding to daily living tasks identified by users.

• Porter module: Reducing physical strain on users by transporting personal items and objects within the care environment—addressing mobility and independence needs.

Ultimately, the 39 FRs categorised as *(i) Possible Now* clearly aligned with these four modules. This structured approach simplified conceptual design and set clear, actionable development priorities for subsequent design phases (CONCEIVE and BUILD). Comprehensive documentation of these FRs and their respective modules is available in the Supplementary Materials accessible on the Miro board (Colle, 2025a).

#### 3.3.6 Define phase evaluation

In the context of the present study, the methodology chosen imposed a significant time commitment on the participants, without any financial remuneration. Therefore, it was essential to ensure that participants found the experience worthwhile and enjoyable. Feedback was collected from participants on each intervention to evaluate their experience. The workshop received good ratings, “Was the workshop worth attending?” scored 4.8/5, and “Do you think we are making progress?” scored 4.82/5, indicating overall satisfaction and perceived progress. Among the participants, four provided positive feedback on the first activity, while one highlighted issues such as insufficient time, unproductive discussions, and technical challenges with the online MIRO platform and connectivity. Key messages included affirmations like “The project feels like a very interesting and worthwhile process” and anticipatory remarks for future sessions. Although some concerns were raised about the limitations of robotics (e.g., “the human role is irreplaceable” or “robot only has limited tasks to perform”), final comments were encouraging, emphasising the potential of the robot to meaningfully assist those in need.

#### 3.3.7 Define phase summary

• Learning Goals: Transform an indeterminate set of user-identified requirements (FR1–98) into clear, prioritised design outcomes.

• Assessment Criteria: Functional priorities were ranked collaboratively through co-design workshops. A consensus-driven clustering process reduced 98 FRs to 39,improving clarity, and feasibility for subsequent development phase.

• Project Constraints: Technological and resource constraints were mapped to each FR and categorized as: “Possible Now”, “Future Development”, or “Possible with Financial Support”. This constraint mapping directly informed which modules were viable within the project’s scope

• Problem Statement: By systematically grouping and ranking FR (1–98) under distinct challenges faced by older adults, the team achieved a defined scope for four actionable modules; Social, Sanitation, Hospitality, and Porter, clarifying the design path and laying the groundwork for the subsequent CONCEIVE and BUILD phases.

### 3.4 Conceive the outline of a solution

The CONCEIVE phase focuses on generating and refining conceptual models that outline potential solutions to the defined problem. During this phase, teams create non-functional, symbolic representations of the design, such as design arguments and service blueprints, which serve as use cases for analysing how different components interact to achieve the intended goals.

#### 3.4.1 Defining the DPs

In alignment with the Axiomatic Design framework (Figure 2), the 39 FRs defined during the DEFINE phase were systematically translated into four modules, represented by their DPs. Each DP provided a concrete conceptual response to clusters of FRs. Thus, the following modules were defined: DP1 (Porter module), DP2 (Social module), DP3 (Sanitation module), DP4 (Hospitality module).

#### 3.4.2 Conceptualisation of modules

**FIGURE 6:**
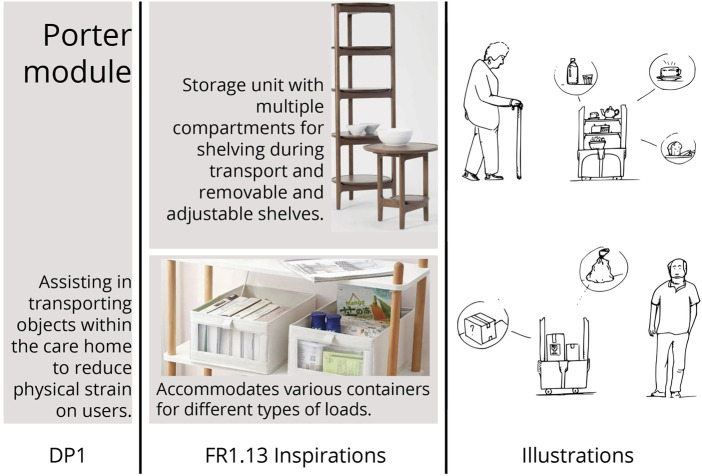
Steps in the design process for the Porter module: defining DP1, sourcing inspiration from existing objects, and developing initial conceptual sketches.

To illustrate the conceptualisation process clearly, this subsection focuses specifically on the Porter module (DP1). Figure 6 depicts the conceptualisation sequence, beginning from DP1 definition, moving through inspiration from existing real-life storage solutions, and concluding with initial conceptual sketches. The core idea of the Porter module stemmed directly from FR1.13, which specified a tray-based transport mechanism suitable for meals and personal items. By referencing existing products and ideas, it helps the team to refine the initial concept into a more mature design. The illustrations on the right quickly establish the core concept, solidifying the idea within the development team.

#### 3.4.3 Co-design workshop with participants

With the easing of COVID-19 restrictions, the research team conducted the project’s first in-person workshop at the care facility. Participants reviewed detailed sketches of each module (DP1–4), supplemented by a concise summary of insights from the earlier UNDERSTAND phase. Using guided co-design techniques, participants provided targeted feedback to refine the conceptual designs, proposing sub-FR (1. x–4. x) tailored to their Personas’ specific needs.

Visual sketches were important to the workshop, chosen specifically for their effectiveness in clearly conveying complex ideas and eliciting rich participant feedback (Chu et al., 2017). For each DP, participants answered structured Wants and Needs (W&N) questions, directly refining module specifications. Examples from participant responses to the Porter module (DP1) included:

• How would your persona interact with this module? “Needs to be practical when wants to drink, needs a beaker if impaired” or “First thing in the morning or late at night, bring coffee or hot chocolate.”

• List some actions for which your persona will use this device. “To hold a meal and a cup of tea from the kitchen to the living room” and “Carrying objects, mobile phone, tablet, remote for stereo, TV, CDs, snacks.”

• What potential problems could arise? What do we need to keep in mind? “Cannot tip over” or “From what sides the users will get or put drinks in the trays?”

Overall, 36 detailed sub-FRs (FR1.1–1.36) were gathered during the workshop and are available in the Supplementary Materials on the Miro board (Colle, 2025b).

#### 3.4.4 Conceive evaluation method

**FIGURE 7:**
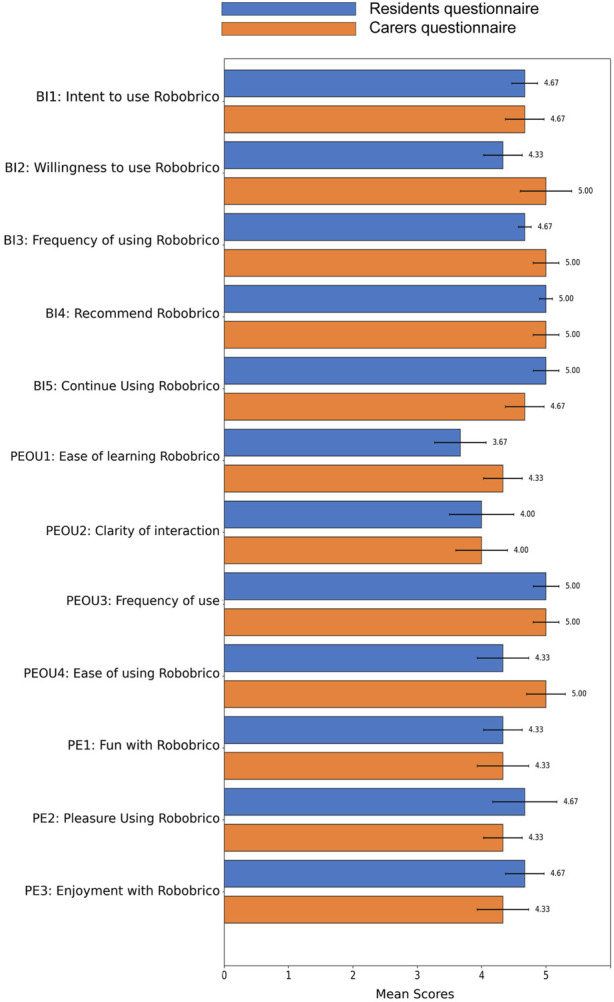
Comparative evaluation of Robobrico acceptance among Residents and Caregivers, reflecting BI, PEOU, and PE. The data indicates a majority of positive acceptance among participants.

Following the conceptualisation workshop, an evaluation was performed to measure participants’ perceived ease of use, behavioural intention to use, and enjoyment of the proposed robot modules. Utilising the Consumer Acceptance Model questionnaire for technology acceptance (Gao and Bai, 2014), participants’ responses were quantitatively captured across three dimensions: Behavioural Intention of Use (BI), Perceived Ease of Use (PEOU), Perceived Enjoyment (PE). Figure 7 summarises the comparative evaluation results between residents and caregivers, reflecting broadly positive attitudes toward Robobrico’s usability and perceived value.

Both resident and caregiver groups expressed positive intentions to engage with Robobrico, with particular optimism regarding future adoption as familiarity increases. Concerns about initial resistance—particularly among elderly users less accustomed to technology—were acknowledged but considered manageable with proper introduction and sustained support. Participants emphasised the critical importance of the robot’s first impression and its introduction to the broader community. They recommended continuous refinement, simplicity, and practicality in the design to ensure widespread adoption. Clear expert support and intuitive user interaction pathways were highlighted as essential for encouraging regular use and sustained engagement.

#### 3.4.5 Conceive phase summary

• Design Models: Conceptual design arguments and early module definitions were created translate FR (1–39) into (DP1–4). These models outlined possible interventions that align with user needs, guiding the direction of subsequent development.

• Conceptual Framework: Using Axiomatic Design principles, within the DR methodology to create replicable and concrete robotic outcomes.

• Blueprint Development: Service blueprints and sketches were produced to visualize how modules would operate within the user environment. These symbolic representations helped the team and stakeholders explore functionality and user interaction pathways before implementation.

• Component Interaction: Co-design workshops and early evaluation exercises clarified how users would engage with specific module features.

### 3.5 Build a solution

The BUILD phase involves constructing a functional prototype to test and refine the design. For hardware, it includes assembling mechanical and electronic components, integrating necessary systems, and ensuring compatibility. Initial prototypes may be low-fidelity for quick validation before refining for durability and performance. Testing during this phase identifies and addresses mechanical, electrical, or integration issues. Additionally, manufacturability considerations ensure a smooth transition to production.

#### 3.5.1 Locomotion module

Robobrico, being a manually reconfigurable robot, requires a practical base to navigate its environment effectively. One fundamental principle of the Axiomatic Design process is to maintain the independence of FRs, as stated by Suh’s Independence Axiom (Suh, 1998). This principle holds that if modifying one DP influences multiple FRs, the design becomes undesirably coupled. Although users did not explicitly request a Locomotion module, their need for autonomous movement and the requirement to keep modules independent implied the necessity for such a component. Consequently, the Locomotion module is defined as DP5. Its primary FRs (5. x–5. y) align with the definitions of AMRs (Siegwart et al., 2011), emphasising both mobility and autonomy. Moreover, because Robobrico is manually reconfigurable, this Locomotion module must be easily and safely replaced by non-specialist operators, with a connection system that ensures interoperability, safety, and integrability (see Modular Robot section). Although this aspect technically belongs to the DEFINE phase, it is describe here to clarify the rationale behind including a dedicated Locomotion module.

**FIGURE 8:**
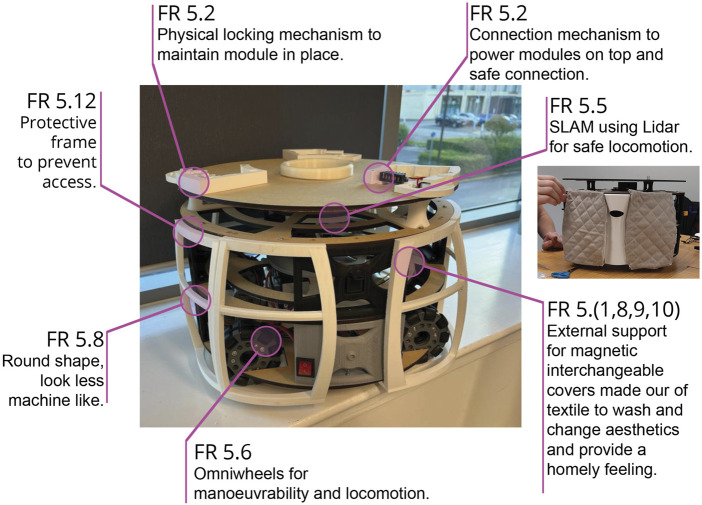
Photograph of an initial complete prototype Locomotion module, providing an overview of the design considerations incorporated, as detailed in Table 4. The design considerations integrated into the final module reflect on participants’ feedback.

In Table 4, FR (5.1–5.16) summarises the inputs from participants during the CONCEIVE phase in response to the W&N questionnaire. The resulting DP (5.1–5.16) reflects the team’s physical alterations to the Locomotion module, based on participants’ feedback, and these DPs were incorporated as design considerations wherever possible in the prototype shown in Figure 8. Spatial measurements and ergonomic guidelines established during the UNDERSTAND phase informed critical DPs. Specifically, the robot’s diameter was set to 45 cm to ensure effective navigation in constrained care environments, while the overall height was defined within the range of 85–95 cm, aligning with ergonomic recommendations for older adults (Salvendy and Karwowski, 2021). The BUILD phase concentrated primarily on detailed development and prototyping of the Locomotion and Porter modules.

**TABLE 4:** FRs (5.1–5.16) and corresponding DPs (5.1-5.16) related to the Locomotion module, grouped into thematic design categories reflecting current or potential physical modifications.

Category	FRs	DPs
Usability & Accessibility	5.1 Easy to clean, wipeable	5.1 Material to cover the robot that is easy to wash and potentially removable
	5.2 Ensure the robot is physically safe to avoid triggering falls, consider physical presence of the robot	5.2 Robot needs to be structurally safe
	5.3 Be aware of impairments	5.23 Will be developed in a future iteration of the project
	5.4 Warning for deaf people using specific sound	5.4 Will be developed in a future iteration of the project
Safety & Navigation	5.5 Avoid bumping into people, walkers, walking sticks, prevent hitting people in the legs	5.5 Smooth way to navigate in different spaces using LIDAR for path planning
	5.6 Stability, potentially using four wheels	5.6 First iteration of Robobrico using four Omni-wheels
	5.7 Light or sound to make users aware of the robot’s presence	5.7 Will be developed in a future iteration of the project
Aesthetics & Personalization	5.8 Homely, round, should not look like a machine	5.8 Considering aesthetics closer to building furniture rather than traditional robotics design, using alternative materials to cover the robot
	5.9 Adaptable to user tastes, such as colour customization (e.g., tenant loves pink), users’ colour choice	5.9 Leave a placeholder for people to add their own preferences in terms of textiles or decorative objects
	5.10 Changeable outlook	5.10 Future iteration can enhance customization
	5.11 Plain colour and calming mood lights (calm colour), mood lights on the robot like in screen savers	5.11 Integration of LEDs into the robot design
Materials & Durability	5.12 Hard surface	5.12 Robot chassis designed to be covered with a hard surface
	5.13 Waterproof	5.13 Electronics and live wiring safely covered by an IP65 electric box
Interaction with Environment & Animals	5.14 Consider smells that dogs like	5.14 Could be developed in a future iteration of the project
	5.15 Consider animals being afraid of being run over	5.15 Could be developed in a future iteration of the project
Module Management	5.16 Practical charging station	5.16 Power button and charging through standard (IEC C13) power socket

The initial prototype of the Locomotion module (Figure 8) satisfied several DPs summarized in Table 4. Drawing from the structural arrangement of the Roboshop off-the-shelf robotic platform (Roboshop, 2025), the Locomotion module incorporated a three-tier configuration: a lower section housing the drivetrain and battery units, a central layer accommodating electronic hardware and processing components, and an upper segment containing Lidar sensors alongside standardized connection interfaces for additional modules.

Custom-developed control software was integrated to operate and coordinate attached modules (Colle et al., 2021), providing autonomous navigation functionalities such as Simultaneous Localization and Mapping (SLAM) and Computer Vision (CV). The robot employed a rudimentary hybrid, goal-based architecture, with the goal of using dynamic management of modules and facilitating adaptive behaviour contingent upon current configurations. Furthermore, a physical interconnection mechanism was implemented to support hot-swappable energy and data transfer between modules, complemented by modular software architecture built upon ROS, enabling dynamic reconfiguration.

**FIGURE 9:**
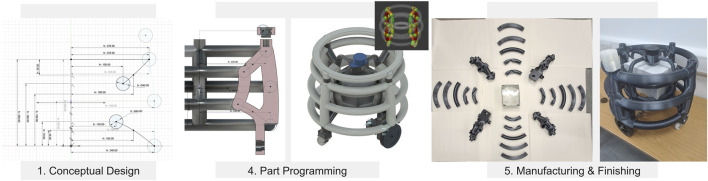
Illustration of the key sequential design steps during the development process of the Locomotion module following Pradel’s et al. framework (Pradel et al., 2018). This final prototype module was designed to enhance safety, robustness and ease of manufacturing.

Following initial testing, the Locomotion module underwent physical design refinement (Figure 9) aimed at enhancing safety, robustness, and manufacturability. Key modifications included the reduction of overall component count to simplify assembly processes, integration of an IP65-rated enclosure to protect electronic systems from environmental factors, and removal of potential safety hazards associated with unsecured fittings. The revised enclosure consolidates both the robot’s control and power management systems into an interchangeable unit, thereby enhancing the modularity nature of Robobrico. In theory, this approach enables consistent control functionality irrespective of variations in the physical attributes of different locomotion host bases. Future iterations will further refine this modular architecture, with the ultimate objective of integrating Explainable AI principles to enhance transparency in decision-making and support the emergent behaviours facilitated by interchangeable robotic modules.

#### 3.5.2 Prototype development using DfAM framework

In the BUILD phase, we adopted the DfAM framework proposed by Pradel et al. (Pradel et al., 2018), represented in Figure 3, to systematically transform our conceptual designs into fully realised hardware. The following subsections detail each stage of this framework as applied to the Locomotion module, covering core development decisions:

• Conceptual Design: Defines functional intent and explores AM-relevant design directions. To reduce the amount of parts and fasteners (bolts, screws) for enhanced platform safety, the team adopted a PM approach to define the chassis’s overall load-bearing requirements and house an IP65-rated enclosure. PM, as illustrated in Figure 9, allows to set up high-level parameters (dimensions of train drive, battery, electronics, Lidar, connection points). Consequently, any dimensional change automatically and updated the 3D model, avoiding extensive manual edits on the geometry of the model. From the outset, Fused Deposition Modelling (FDM) was chosen due to budget constraints and the need to print large parts reliably, which steered the material selection (carbon-reinforced Polylactic Acid (PLA)) towards affordability, ease of print and sufficient mechanical strength.

• Embodiment Design: Develops structural layout and component relationships with AM constraints in mind. With Fusion 360 (Autodesk, 2025) used for PM we created cavities and extrusions to embed the IP65 enclosure within the chassis structure. This facilitated integrated power and signal routing, minimising external wiring. Meanwhile, a large-bed Raise3D printer (Raise, 2025) was secured to handle the chassis’s overall footprint. Recognising the risk of failed builds with oversized prints, the chassis was subdivided into multiple sections, each sized and oriented to mitigate warping or support-related failures.

• Detail Design: Finalises features, dimensions, and tolerances using AM design rules. Within each subdivided component, wall thicknesses were doubled to enhanced robustness, balancing faster print speeds with the structural demands of a load-bearing potential modules. Overhangs were reduced to avoid excessive supports, thus speeding up printing and assembly. Components such as wheels, electronics, and Lidar mounts were refined in the 3D model and integrated to ensure proper fit. This phase particularly leveraged Japanese carpentry joints (Brown, 2014) for interlocking surfaces, specifically a right angle and mortise splice, maintaining chassis integrity without relying on nails or metal fasteners.

• Part Programming: Generates machine instructions and optimises print parameters. Once geometry was finalised (Figure 9, each of the 25 sub-parts was prepared for printing through slicing and tool path generation, tuned to the mechanical properties of carbon-reinforced PLA. Print parameters, layer height, infill, support density, were optimised to handle bridging and deliver strong interlocks. Any failed test prints led to small modifications, typically at the embodiment or detail design level (e.g., adjusting joint shapes or layer thickness, print orientation).

• Manufacturing & Finishing: Executes printing and post-processing; validates part quality. All segments were 3D-printed, captured in the photograph in Figure 9, then assembled using hot glue for sealing. The Japanese carpentry joints provided both strength and flexibility, enabling the chassis to withstand stress without additional fasteners. Final touches included light sanding to smooth visible seams.

#### 3.5.3 Porter module

Applying this structured design method provided a clear framework for each stage of the Locomotion module’s development, ensuring traceability in decisions related to FRs, engineering constraints, and user objectives. In subsequent iterations, integrating the feedback loops presented by Pradel et al. (Pradel et al., 2018) would allow newly acquired insights—such as user test data, material properties, or manufacturing constraints—to inform earlier phases, thereby enhancing both the reliability and adaptability of the module.

**FIGURE 10:**
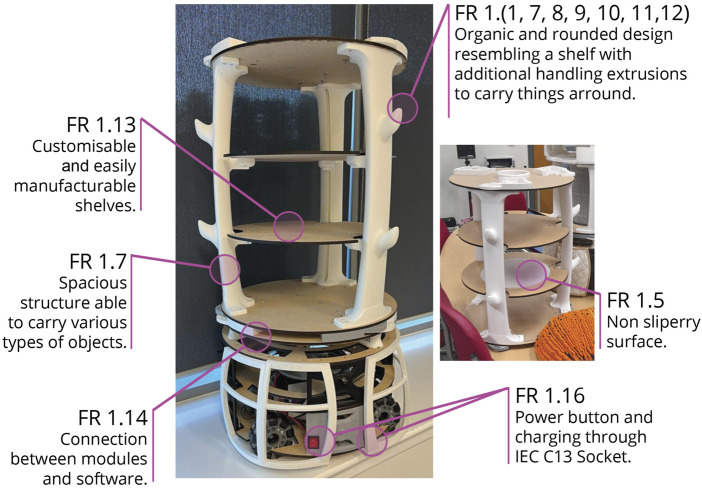
Overview of design considerations for the robot, incorporated into the Locomotion and Porter module, as detailed in Table 5, which summarises the inputs from participants in response to their Wants and Needs (W&N).

In Table 5, FR (1.1–1.16) summarises the inputs from participants during the CONCEIVE phase in response to the Wants and Needs (W&N) questionnaire. The resulting DP (1.1–1.16) reflects the team’s physical modifications to the Porter module based on this feedback, and these DPs were implemented wherever possible given the team capacity as described in Figure 10.

**TABLE 5:** FRs (1.1–1.16) and corresponding DPs (1.1-1.16) related to the Porter module, grouped into thematic design categories reflecting current or potential physical modifications.

Category	FRs	DPs
Usability & Accessibility	1.1 Ensuring the robotic platform is user-friendly, adaptable, and focused on improving everyday tasks safely	1.1 Create a simple and approachable aesthetic, similar to what people are used to in their everyday lives
	1.2 Assisting elderly users with daily tasks that might otherwise be difficult	1.2 Identified as a useful feature for the Porter module, defining with users what might be useful to carry around
Safety & Navigation	1.3 Secure, lidded containers for handling hot liquids (e.g., tea, coffee)	1.3 Focus on carrying cold liquid as a start
	1.4 Stability when handling fragile or hot items	1.4 Need a stable Locomotion module to carry the Porter module
	1.5 Non-slip surfaces to prevent accidents	1.5 Create trays with non-slippery surfaces
	1.6 Direct drink dispensing to avoid spills and accidents	1.6 Additional supply of dispensers on the module in a future version
Practicality	1.7 Transporting laundry 1.8 Transporting groceries 1.9 Delivering items like newspapers 1.10 Carrying personal items 1.11 Carrying meals 1.12 Carrying rubbish	1.7 Create a large enough module with space for baskets of laundry and an adaptable tray system to accommodate different objects
Aesthetics & Personalization	1.13 Customizable shelves for flexible usage	1.13 Removable shelves from the core of the Locomotion module
Module Management	1.14 Careful management of modules for enhanced functionality	1.14 Development of the software to control the modules and connection mechanism
User Accessibility	1.15 Voice recognition for easy interaction	1.15 Will be developed in a future iteration of the project
	1.16 Large-button remote controls for users with impairments	1.16 Will be developed in a future iteration of the project

The Porter module was developed using the same methodology used for the Locomotion module. Most of its components were 3D printed, while the shelves, base, and top were fabricated by laser-cutting plywood, and the 3D printed parts were integrated with the laser-cut elements.

#### 3.5.4 Sanitation and social module

**FIGURE 11:**
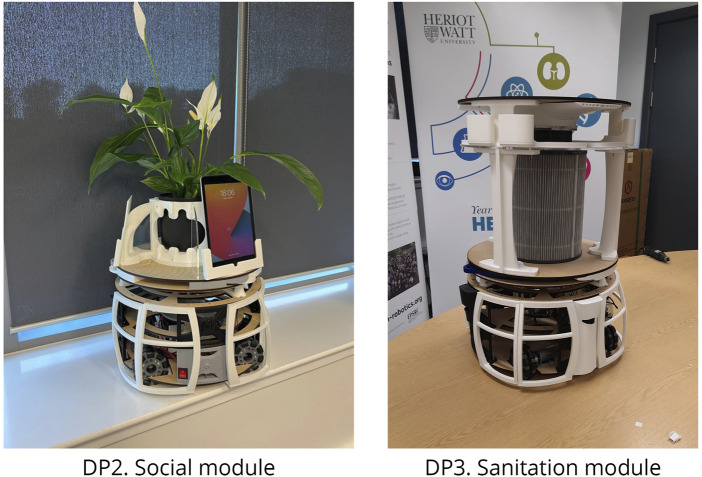
Photographic images of different robot configurations, demonstrating the final design outcomes of Robobrico, for the Social module (DP2) and the Sanitation module (DP3).

Using the methods previously described, the team developed two additional modules, illustrated in Figure 11. The Sanitation module (DP3) integrates a HEPA filter cartridge combined with a high-capacity GPU fan, providing effective suction capabilities. It also incorporates an air-quality sensor, enabling future autonomous monitoring of environmental conditions within care settings.

The Social module (DP2) integrates functionalities that support both interpersonal interactions and digital connectivity. Designed to accommodate multiple electronic tablets for video conferencing or teleoperation, it features compartments for personal belongings, such as spare glasses or keys, and space for decorative items like plants to promote familiarity within care environments. The partially open and flexible design encourages personalisation by users, aligning with Gibson’s concept of ’taming technology’, whereby technology is adapted to users’ individual preferences and routines.

Less detail is provided in this section, because the primary technical focus of the BUILD phase concentrated on the Locomotion and Porter modules, whose development processes are documented above. However, both the Sanitation and Social modules were created using the same methodology.

#### 3.5.5 Build phase summary

• Prototype Creation: Initial low- and high-fidelity prototypes of the modular robotic system were developed to test functionality and validate user-informed design decisions.

• Solution Implementation: Key basic systems such as SLAM, computer vision, and modular control architectures were implemented to support autonomous operation and modularity.

• System Development: A ROS-basic software framework was built to manage in future version dynamic module configurations, including communication protocols and energy/data interconnects between modules.

• Material Construction: Using DfAM, components were produced via FDM 3days printer, using carbon-reinforced PLA, balancing cost, strength, and ease of fabrication. Parametric modelling enabled rapid iteration, while Japanese carpentry joints allowed screwless, robust assembly.

• Functional Design: Modules were designed for safety, accessibility, aesthetics, and real-world usability, incorporating ergonomic standards and feedback from participants.

### 3.6 Test a solution

In the TEST phase of the DR process the focus is on evaluating the effectiveness of the developed solution within its intended context. This phase involves collecting data through various methods and studies to examine the performance of the solution and gather feedback. The goal is to determine whether the practical goals have been met and to test the theoretical design arguments. The testing process is iterative, allowing for rapid prototyping and feedback collection, which informs further refinements of the solution.

The final evaluation was conducted through an on-site workshop at the care partner’s facility, where participants tested and provided feedback on the Robobrico modules developed in the BUILD phase. Given the project’s progression, no immediate design modifications were applied; rather, insights were collected to guide future iterations, commencing at an appropriate point within the DR cycle (see Figure 1).

#### 3.6.1 Design iteration

Participant feedback was documented without immediate implementation, focusing instead on recording insights for future development. Table 6 summarises participant evaluations for the Locomotion and Porter modules. Participants positively noted the compactness and practicality of the Locomotion module, suggesting improvements related to aesthetics and protective features for exposed electrical components. For the Porter module, functionality received positive reviews, with recommendations focusing on stronger materials and improved security for personal items.

**TABLE 6:** Comparison of user feedback on the Locomotion and Social modules, grouped by thematic design aspects including form factor, materials, storage, functionality, and user concerns.

Theme	Locomotion module	Social module
Form Factor & Aesthetics	Not a cumbersome, solid unit Fits most rooms (height, dimensions) Non-threatening appearance Can be decorated (e.g., fairy lights)	Options for a personalised look Plastic material raises sturdiness questions No raised edge: items may roll off
Materials	Plywood is porous – consider sealing Material-only base might expose internal components Electrical compartments should be fully enclosed	Consider reinforced plastic or a cover Fruit bowl featured in render (absent in prototype)
Storage & Organisation	Primarily open design Add a small lip to prevent items from rolling off	Drink holders Compartments for keys, phone, remoteetc. Space for books, magazines, and newspapers Potential for a drawer or tray-holder
Features & Functionality	Some older adults may not use tablets/smartphones	Easy inclusion of personal photos Options for holding dog treats or children’s toys (e.g., Lego) Option for a phone/tablet stand
Concerns & Suggestions	Ensure users cannot access internal electronics	No ledge: risk of items falling Consider reinforcing plastic or using a cover

#### 3.6.2 Usability and functionality assessment

**FIGURE 12:**
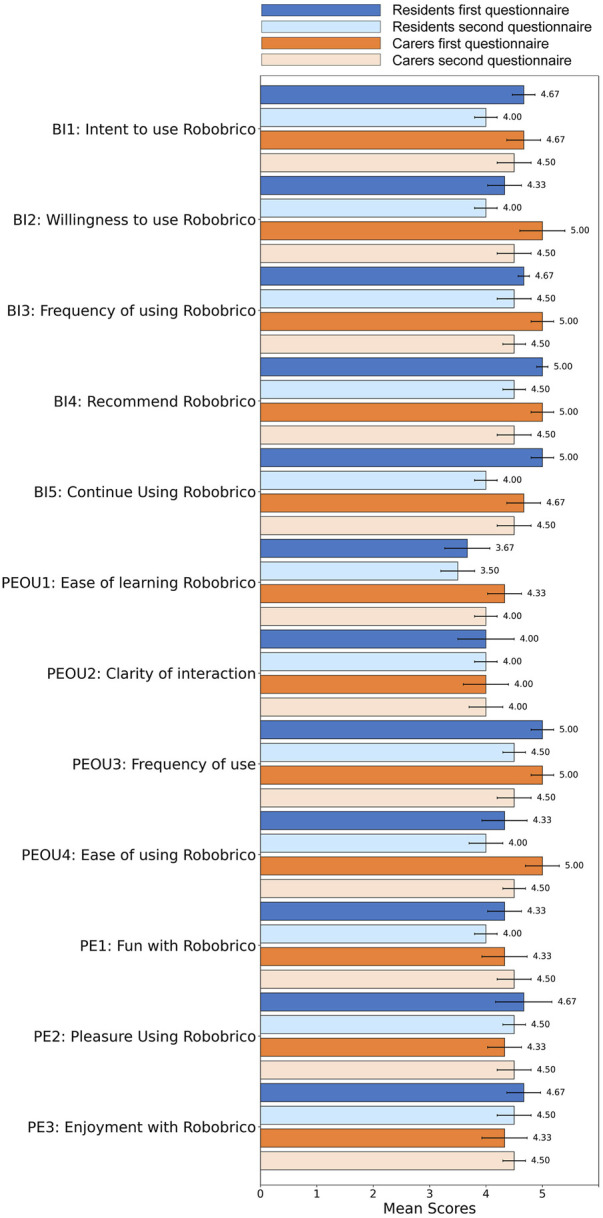
Comparative analysis of participant scores for ease of use and intention to use between CONCEIVE and TEST phases indicating positive perceptions across both evaluations.

To evaluate participants’ evolving perception of Robobrico, the same Consumer Acceptance Model questionnaire used during the CONCEIVE phase was administered again during the TEST phase. Comparative analysis between these two stages is illustrated in Figure 12.

Participants’ responses indicated stable and generally positive perceptions across both evaluations. Specifically, scores related to Behavioural Intention (BI) remained consistently high, signifying sustained interest in using Robobrico. Notably, slight improvements in ease of learning (PEOU1) and overall ease of use (PEOU4) were observed, suggesting enhanced familiarity and comfort among participants with the system over time. Scores related to enjoyment (PE1, PE2, PE3) continued to reflect strong positive engagement.

#### 3.6.3 Kano analysis for functionality prioritisation

**FIGURE 13:**
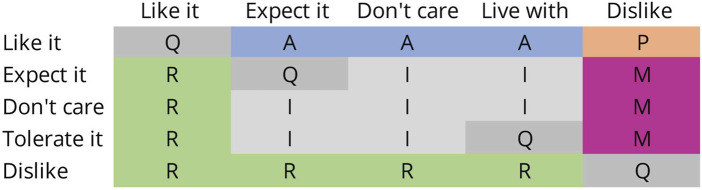
The Kano evaluation table, categorizes product features based on user satisfaction and dissatisfaction responses. The rows represent customer reactions when a feature is present (functional), while the columns indicate reactions when a feature is absent (dysfunctional).

We performed a Kano analysis (Zhang et al., 2024; Materla et al., 2019) to assess the impact of various functionalities and prioritise user preferences. The Kano model categorises requirements into five groups, “Attractive (A), One-dimensional (O), Must-be (M), Indifferent (I), and Reverse (R) (with Q for Questionable)”, as shown in Figure 13 (Yang et al., 2021; Zhang et al., 2024). This model distinguishes between basic expectations and features that improve satisfaction. In healthcare, for example, it helps identify essential services such as safety and hygiene, as well as additional features like personalised attention that can increase patient satisfaction. For a robotic platform like Robobrico, the Kano analysis ensures that fundamental functionalities are present while also highlighting opportunities to delight users with additional capabilities.

Results from the Kano analysis highlighted clear user preferences. All participants categorised the Porter module as a one-dimensional feature, indicating its critical role, as improvements directly correlate with increased user satisfaction. Similarly, half of the participants rated the Sanitation module as one-dimensional, underscoring its substantial importance. Therefore, future design iterations should prioritise enhancements in both Porter and Sanitation modules to effectively meet core user requirements.

In contrast, the Social module was predominantly classified as an attractive feature. Its presence significantly improves satisfaction but its absence does not result in dissatisfaction, suggesting that while beneficial, it remains non-essential. Consequently, future developments should consider the Social module as a value-added component, secondary to the essential functionalities provided by the Porter and Sanitation modules.

#### 3.6.4 Test phase summary

• Performance Evaluation: Two questionnaires, plus comparative stages (CONCEIVE vs TEST) assessments, measured how the Locomotion and Porter modules met user needs.

• Feedback Collection: Structured feedback was gathered via workshops and thematic comparison of modules, revealing practical suggestions for form factor, materials, safety, and storage—directly informing future development.

• Validation Process: A Kano analysis was conducted to prioritize user-valued features, distinguishing critical modules (e.g., Porter, Sanitation) from optional enhancements (e.g., Social), ensuring alignment with stakeholder satisfaction.

• Iterative Testing: Although no additional refinements were implemented at this final project stage, findings will guide future iterations, ensuring an adaptive approach to module development.

### 3.7 Present a solution

In the PRESENT phase of the DR process, the focus is on communicating the design solution to key stakeholders, including both practical and theoretical outcomes. This phase involves demonstrating how the solution addresses the initial problem and satisfies stakeholder needs through presentations, reports, and publications. Communication should include evidence of the effectiveness of the solution, the design process, and insights gained throughout the project. This process is beneficial for collecting evidence to secure funding from grants or private sources because the information gathered is well-organised and thoroughly documented.

In the context of this study, this paper itself represents the PRESENT phase by offering a detailed description of the methodologies used, insights gained, iterative prototyping outcomes, and evaluations. For researchers, roboticists, and designers, this documentation serves as a replicable framework illustrating how systematic co-design, iterative feedback, and empirical validation can be integrated within a structured DR and Axiomatic Design methodology. This paper thus provides a blueprint for systematically gathering and translating user-identified FRs into tangible, modular, and adaptable robotic solutions.

However, it is important to emphasise that the PRESENT phase does not denote the completion of the design process. As illustrated previously in Figure 1, the DR process is inherently iterative and dynamic, continually cycling through stages of user engagement, ideation, prototyping, testing, and refinement. Design, particularly in complex, user-centred fields such as social robotics, must be understood as an evolving collaboration between developers and end-users rather than as a finite project.

This aligns with the concept of a “Liquid” design process described by Pradel et al. (Pradel et al., 2019), emphasising flexibility, adaptability, and continuous improvement. With the ongoing advancements in DfAM, Design for X, and emerging frameworks like Design for Circularity (Moreno et al., 2016), the possibilities for continual development and adaptation are expanding. Robotics, as an interdisciplinary domain combining hardware, software, and social interaction, will particularly benefit from these evolving methodologies, enabling platforms to better align with changing user needs.

Ultimately, this ongoing design cycle fits into the broader market trend of Mass Individualisation (Sikhwal and Childs, 2021), where manufacturing processes increasingly prioritise customisation and personalised user experiences over mass production. The Robobrico project embodies this philosophy, demonstrating how structured, iterative, and user-driven methods can produce highly adaptable and meaningful robotic solutions.

#### 3.7.1 Present phase summary

• Final Reporting: This paper serves as the public-facing documentation of methods, prototypes, and outcomes, providing a reference for future studies and funding opportunities.

• Impact Assessment: By capturing both practical and theoretical results, the project’s value to stakeholders, funding bodies, and end-users is demonstrated, emphasising the significance of iterative design and co-development.

• Project Presentation: Presentations, publications, and Supplementary Materials communicate the systematic development process, encouraging broader adoption and adaptation of this methodology in social robot design.

## 4 Discussion and future work

In conclusion, this work establishes a robust foundation for a co-design methodology aimed at developing a modular SAR platform. Despite a limited number of participants—including care specialists, managers, and residents—the study generated substantial quantitative and qualitative data that supported the seven-phase, step-by-step approach demonstrated with the Robobrico platform. This method enables rapid, measurable design iterations, supports fast prototyping, and lays the groundwork for future manufacturing.

Participants feedback was positive, with sustained motivation observed both during and after the COVID-19 period. The collaborative process facilitated the systematic capture of individual experiences within the care sector and translated these insights into actionable guidance on how automation may support daily activities. Until ubiquitous robotics become a reality, interim tools that simplify user interactions and enhance usability remain essential, particularly for non-technical audiences.

Furthermore, the methodology provided a framework for constructing a tangible robotic system through the co-design process. Using the flexibility of DfAM, a functional device was developed that enabled users to assess the impact of technology in their environment. The results demonstrate that, even with limited resources, combining DfAM with inspiration from everyday objects can facilitate the creation of DIY systems that offer practical functionality at low costs.

Although the process required considerable effort from non-remunerated participants, the valuable insights obtained justified this commitment. However, the findings suggest the methodology could benefit from further refinement to reduce participant burden. The limited sample size used in this initial study effectively managed workload and allowed for the method’s preliminary evaluation. With the data and feedback collected, future studies can confidently plan for larger participant samples, supported by appropriate budgets and resources. Overall, the results affirm that a comprehensive co-design approach is essential for accurately understanding user requirements and effectively guiding SAR development, thus facilitating further refinement and broader applicability in subsequent research.

### 4.1 Future work

The current study represents an initial step in a broader development process for modular robotic solutions in elderly care. A central methodological component was the application of the “Day in the Life” approach, producing detailed usage scenarios that closely reflect daily experiences within care environments. These scenarios subsequently informed the definition of FRs and their translation into DPs. Future research will build upon this foundational work by conducting large-scale validation studies to identify commonalities across scenarios, FRs, and DPs. Such studies will facilitate better prioritisation and targeted development of robotic modules suitable for diverse care contexts both within and beyond Scotland.

Additionally, the present research primarily relies on qualitative participant feedback. To strengthen and objectively validate design outcomes in subsequent iterations, incorporating quantitative assessment metrics such as Task Completion Rates, System Usability Scales, and Time-on-Task analyses is recommended. A larger participant sample, extended engagement periods, and iterative refinement of robot prototypes will be pursued to enable robust quantitative evaluation and improved generalisability of findings.

This research adopted Axiomatic Design principles to systematically structure requirements and design solutions. While the Independence Axiom guided the mapping of each FR to a unique DP, thereby minimising complexity—the Information Axiom promoted the selection of designs with minimal information content to enhance efficiency and reliability. Future investigations will extend beyond the identification of FRs and DPs, examining the probabilistic approaches inherent in the Design Matrix. Specifically, forthcoming studies will explore how categorising designs into uncoupled, decoupled, or coupled systems influences system efficiency, reliability, and manufacturability. Emphasis will also be placed on minimising information content to simplify rapid prototyping through DfAM.

Moreover, additional research funding from the EMERGENCE Network (Emergence, 2025) has already been secured to further investigate practical care issues such as hydration management in care homes. Leveraging the iterative nature of the established design methodology, future projects will also explore the integration of complementary methods and align with ongoing research efforts in ethics, behavioural design, and broader HRI domains, as outlined by Axelsson et al. (Axelsson et al., 2022).

Finally, the proposed methodology holds significant potential not only within academia but also for practical implementation within the private sector. Unlike highly complex humanoid robots, modular robots developed through this approach can effectively address specific care tasks, achieving robust functionality at reduced complexity and lower costs. By emphasising semi-autonomous operation, community-driven upgrades, and user engagement, the Robobrico framework aims to create affordable, maintainable robotic solutions specifically suited to sectors facing financial constraints. Future commercial developments could thus foster sustainable adoption, encourage community ownership, and promote wider accessibility to robotic care solutions.

## Data Availability

The raw data supporting the conclusions of this article will be made available by the authors, without undue reservation.
