# Supplementary material for: Co-design methodology for rapid prototyping of modular robots in care settings
Source: Front Robot AI. 2025 May 22;12:1581506. doi: 10.3389/frobt.2025.1581506 (PMC12137090; doi:10.3389/frobt.2025.1581506)
Supplement: Supplementary file 1 [file DataSheet1.pdf]

The accompanying framework and supplementary materials are made available for academic use via the Miro platform at the following link: https://miro.com/app/board/uXjVLhsta0s=/?share_link_id=698075600891.

This work is released under the MIT License, permitting free use, modification, and distribution provided that appropriate attribution is given. These resources are provided to facilitate replication, further study, and the development of derivative works in an academic context, in accordance with the terms of the MIT License.
